# Recovery Experiences Protect Emotionally Exhausted White-Collar Workers from Gaming Addiction

**DOI:** 10.3390/ijerph191912543

**Published:** 2022-10-01

**Authors:** Meng Xuan Zhang, Long W. Lam, Anise M. S. Wu

**Affiliations:** 1 Department of Medical Humanities, School of Humanities, Southeast University, Nanjing 211189, China; 2 Department of Management and Marketing, Faculty of Business Administration, University of Macau, Taipa, Macau, China; 3 Centre for Cognitive and Brain Sciences, Institute of Collaborative Innovation, University of Macau, Taipa, Macau, China; 4 Department of Psychology, Faculty of Social Sciences, University of Macau, Taipa, Macau, China

**Keywords:** gaming addiction, emotional exhaustion, recovery experiences, relaxation, Chinese workers

## Abstract

Gaming addiction and its negative consequences have attracted public concern, but relatively little is known regarding its impact on adult workers. This study aims to test the association between gaming addiction and emotional exhaustion as well as the protective effect of recovery experiences on such an association among white-collar workers. We recruited 205 working adults (female = 58%) who voluntarily completed an online questionnaire. The results showed that male and younger workers were more vulnerable to gaming addiction. Emotional exhaustion was significantly and positively associated, while two (i.e., relaxation and control) of the four recovery experiences were negatively correlated with gaming addiction. Control experience had an indirect effect on gaming addiction via emotional exhaustion. Moreover, relaxation experience buffered the risk effect of emotional exhaustion on gaming addiction so that the effect is less pronounced at high levels of relaxation. Interventions are suggested to raise workers’ awareness of effective recovery experiences (especially for control and relaxation) and to facilitate their recovery opportunities.

## 1. Introduction

When gaming has been viewed as the most addictive type of Internet use [1], and due to the high prevalence of computer and mobile devices, increasing attention has been paid to the addictive and problematic use of electronic games. Gaming addiction, involving online and/or offline games, is now considered a psychiatric condition and listed in the International Classification of Diseases 11th Revision. Major symptoms of gaming addiction include loss of control over gaming, preoccupation with games, and keeping gaming despite impaired consequences [2]. The global prevalence of gaming addiction was around 3.05% between 2009 and 2019, and the trend has been increasing [3]. Considering its high prevalence and numerous adverse consequences on both physical and mental health (e.g., depression and sleep problems) [4,5,6], a bulk of research has been conducted to uncover risk/protective factors and underlying mechanisms of gaming addiction in different populations [7]. This study aims to examine not only the risk factors of gaming addiction but also the protective effects of recovery experiencers against gaming addiction in working adults, who are at risk but have been neglected in prior research.

**Figure 1:**
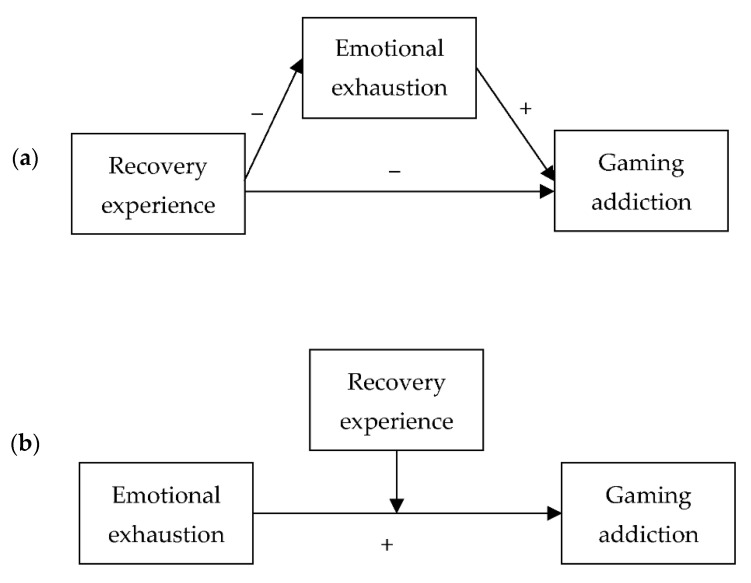
(**a**). The proposed mediation model. (**b**). The proposed moderation model.

Most extant studies on gaming addiction were conducted based on student samples [8,9], and little research has been conducted among working adults. The lack of research here is somewhat surprising given the high prevalence of other addictive behaviors (e.g., smoking and drinking) in this population group [10,11]. To our best knowledge, one study examined the prevalence of gaming addiction among working adults using the criteria for Internet gaming disorder proposed by the American Psychiatric Association [12]. The authors found that 14.1% of the sampled Korean full-time workers were at risk of gaming addiction [13]. Another research illustrated that stress was a risk factor for Internet gaming disorder in Chinese working adults [14]. To enhance our understanding of gaming addiction among working adults, this study examines its association with emotional exhaustion, an adverse emotional outcome due to stress at work. To recover from stress due to work, one can either stop the depletion of resources or gain additional resources from off-work activities. This study thus investigates whether recovery experience, defined as the processes in which individuals can restore the level of their functional systems to the normal or the pre-stress condition [15], will play any protective role against gaming addiction. Figure 1a,b show the mediation and moderation models we proposed. 

Emotional exhaustion generally refers to the feelings of being empty due to chronic work-related stress, which is the core component of burnout [16,17,18]. It is well documented as a general risk factor for mental health and well-being among working adults [19,20]. Most research has reported the negative consequences, including substance addictions, of emotional exhaustion in various working populations (e.g., teachers, mental health professionals, and physicians) [21,22,23,24]. The potential relationship between emotional exhaustion and Internet addiction was also discussed in previous studies [25,26,27]. Specifically, when individuals are emotionally exhausted, they tend to use maladaptive coping strategies, such as drinking and gaming, to lower their stress levels [28]. As such, their tendency to be addicted to electronic games would also increase. Therefore, we hypothesize that there is a positive association between emotional exhaustion and gaming addiction (H1). 

On a daily basis, workers often spend their leisure on various activities (e.g., listening to music and exercises) to recover from their work-related efforts. According to the Conservation of Resources Theory [29], after individuals deal with the challenges and stress at the workplace, their physical and mental efforts will deplete their resources, so the latter must be restored in some ways (e.g., having a big meal) to avoid adverse consequences [30]. Recovery experience refers to the processes that, during a stressful condition, allow individual functional systems to return to their pre-stress levels [31]. Therefore, recovery is also an indicator of a decrease in physiological strain [15]. According to the Effort-Recovery Model [31], recovery experiences comprise four different approaches, namely psychological detachment (i.e., disengaging oneself mentally from work), relaxation (i.e., a state of low activation and high positive affect), mastery (i.e., feeling competent in off-job activities), and control (i.e., a general sense of control over events in their leisure time). Prior research has shown the effectiveness of these four approaches in replenishing one’s resources to vary across situations [15]. Despite their different effectiveness, recovery experiences generally provide an opportunity for individual functional systems to recoup energy and hence are assumed to exert a direct protective effect against detrimental consequences of strain. If the recovery processes are hindered, it will undermine well-being, and emotional exhaustion surfaces [32,33]. Poulsen and his colleagues provided empirical support that recovery experiences, especially psychological detachment and relaxation, were protective factors against burnout among cancer workers in Queensland [33]. The present study, therefore, hypothesized a negative relationship between recovery experiences and emotional exhaustion (H2). 

The Effort-Recovery Model also states that successful recovery experiences not only recoup resources spent to meet work demands but also generate new internal resources (e.g., energy, self-efficacy, and positive mood), which can be utilized during further stress coping and well-being enhancement [15]. Previous studies showed that recovery had an influence on the relationships between job-related strains and well-being [34,35]. A recent study also showed that a higher level of recovery experiences (in terms of control, relaxation, and mastery) was significantly associated with a lower level of depression and anxiety symptoms in doctors during the COVID-19 pandemic [36]. We hence speculate that workers with a high level of recovery experience will be less likely to develop psychiatric disorders, including addictions. To our best knowledge, there is no existing research that tested the association between recovery experiences and any addictive disorders. Gameplay, as one of the highly accessible and economical entertainments in modern society, is often adopted as a coping response to work stress [37], but it may not provide effective recovery experiences to make up for the work demands and generate additional resources for well-being growth as well as against mental disorder (e.g., addiction) development. Therefore, this study is the first to propose this hypothesis: there is a negative relationship between recovery experiences and gaming addiction (H3). 

This present study aims to first examine the associations between emotional exhaustion, recovery experience, and gaming addiction in white-collar workers. Specifically, three hypotheses are proposed: a negative association between emotional exhaustion and gaming addiction (H1), a negative correlation between emotional exhaustion and recovery experiences (H2), and a positive association between recovery experiences and gaming addiction (H3). Furthermore, based on the Effort-Recovery Model, we further propose two unique mechanism(s) for how recovery experiences reduce gaming addiction. First, we expect recovery experiences’ indirect effect on gaming addiction mediated by lowering emotional exhaustion (H4). Second, we expect recovery experiences to buffer the positive relationship between emotional exhaustion and gaming addiction. To be specific, recovery experience is hypothesized to moderate the relationship between emotional exhaustion and gaming addiction so that the relationship is less negative at high levels of recovery experience (H5). By uncovering the risk of emotional exhaustion on gaming addiction, our study shed light on the protective effects of recovery experiences against gaming addiction and even emotional exhaustion in white-collar workers. This study will also identify further directions for studying the protective roles of recovery experience on other addictive behaviors based on the Effort-Recovery Model. The practical insight of this study reveals the effectiveness of recovery experiences regarding preventive interventions for working adults’ gaming addiction.

## 2. Materials and Methods

### 2.1. Participants and Procedures

Employees with at least 6 months of work experience were recruited from So-jump (Wen Juanxing), a crowdsourcing platform for surveying white-collar working adults in China. Two hundred and five participants, of which 58.0% were female, and 69.3% were aged 21–35 years, voluntarily completed the questionnaire. According to the suggestion from Cohen [38] for multiple regression, our sample size was deemed sufficient in terms of statistical power and effect size. The majority of the participants had received a bachelor’s degree (76.6%), and their average work hours were 45 h (SD = 10.39) per week in the last three months. All the participants provided their consent to participate in this study before filling out the questionnaire. They were informed of their right to withdraw from the study at any time without any punishment. Ethics approval was obtained from the ethics committee of the affiliated department of the corresponding author.

### 2.2. Measures

#### 2.2.1. Gaming Addiction

Gaming addiction was measured by a 7-item short form of the Gaming Addiction Scale [39]. The scale includes 7 criteria of gaming addiction, which are salience, tolerance, withdrawal, relapse, conflict, mood modification, and problems. It was assessed with a 5-point Likert scale (1 = never to 5 = very often) with the sample item “Did you spend increasing amounts of time on games?”. A higher total score suggested a higher level of gaming addiction. The internal consistency of this scale was 0.82 in this study.

#### 2.2.2. Recovery Experiences

The Recovery Experience Questionnaire was used to measure those processes of recovery from job stressors [15]. It consists of four subscales, psychological detachment (e.g., “I distance myself from my work.”), relaxation (e.g., “I use the time to relax.”), mastery (e.g., “I do things that challenge me.”), and control (e.g., “I decide my own schedule.”). All the responses to those items were made on a 7-point Likert scale (1 = totally disagree to 7 = totally agree). The reliabilities of these four subscales were 0.88, 0.70, 0.72, and 0.74, respectively, in this study.

#### 2.2.3. Emotional Exhaustion 

Emotional exhaustion was assessed by the 4-item emotional exhaustion scale in the Maslach Burnout Inventory [16]. The participants responded to the items (e.g., “I feel emotionally drained from my work.”) on a 7-point Likert scale (0 = Never to 6 = Everyday), and a higher total score represented more severe emotional exhaustion. The reliability of this scale was 0.89 in this study. 

#### 2.2.4. Demographics 

The participants reported their sex (1 = male and 2 = female), age (1 = 20 years old or younger to 8 = 51 years old or above), educational level (1 = primary school education to 6 = graduate or above), and work hours in the survey.

### 2.3. Data Analysis

According to the procedures proposed in previous research [40,41], no specific outlier was observed in the data set. We then tested the common method bias of all the main variables based on Harman’s single factor test by exploratory factor analysis (EFA) in SPSS 26 (SPSS Inc., Chicago, IL, USA) [42]. According to this technique, the cross-sectional model is less susceptible to the common method bias if there is no factor accounting for more than 40% of the variance of all measurement items. All the other statistics, including descriptive and correlational analyses, were also conducted in SPSS 26. Given that emotional exhaustion and gaming addiction were positively skewed, bootstrapping approach was adopted for mediation and moderation testing because it is less susceptible to violation of the normality assumption [43,44]. The mediating and moderating tests were examined with PROCESS in SPSS. In mediation analysis for the testing of the indirect effect of recovery experiences, recovery experiences and gaming addiction was treated as the independent and dependent variable, respectively, with emotional exhaustion as the mediator based on a partial mediation model. In moderation analysis for the buffering effect of recovery experiences, emotional exhaustion was set as the independent variable, while gaming addiction was the dependent variable. The effect of each of the four recovery experiences was examined individually, with the main effect of the other three types controlled for. We also controlled for the effects of significant demographic correlates (i.e., sex and age) in our mediation and moderation testing. Standardized regression coefficients were estimated with 95% confidential intervals (CI) using the bias-corrected percentile method with 10,000 bootstrapped samples.

## 3. Results

### 3.1. Common Method Bias

All the items of the three variables (i.e., gaming addiction, emotional exhaustion, and recovery experience) were examined with EFA. The unrotated and rotated first factor accounted for 20.52% and 15.37% of the total variance, respectively, which was highly below the threshold of 40%. Therefore, common method bias was not shown in this study.

### 3.2. Preliminary Analyses 

Table 1 shows the bivariate correlation among all measured variables. Regarding demographic effects, age and sex were the significant correlates of gaming addiction (r = −0.18 and −0.19, *p* < 0.01). Significant and negative correlations were also found between age and detachment (r = −0.23, *p* < 0.001), sex and mastery (r = −0.16, *p* < 0.05), as well as work hour and control (r = −0.16, *p* < 0.05).

### 3.3. Bivariate Correlations

As shown in Table 1, emotional exhaustion was significantly and positively correlated with gaming addiction (r = 0.24, *p* < 0.001), supporting H1. Both mastery and control experiences had a significant and negative relationships with emotional exhaustion (r = −0.14, *p* < 0.05 and r = −0.41, *p* < 0.001, respectively) whereas the relationship between relaxation and emotional exhaustion was marginally significant level (r = −0.13, *p* = 0.07). Unexpectedly we found detachment to be positively associated with emotional exhaustion (r = 0.15, *p* < 0.05). Therefore, H2 was only partially supported. Finally, only relaxation and control, but not detachment and mastery, had significant negative relationships with gaming addiction (r = −0.16 and −0.18, *p* < 0.05). Thus, H3 was also partially supported. 

### 3.4. Testing the Indirect and Buffering Effects of Recovery Experiences

The major results of mediation analyses are displayed in Table 2. While there is no significant direct effect of recovery experience on gaming addiction, one of the four experiences, the control experience, has a significant, indirect effect on gaming addiction via emotional exhaustion (β = −0.128, 95% CI [−0.241, −0.034]). Unexpectedly we found a weak, positive indirect effect of detachment on gaming addiction (β = 0.031, 95% CI [0.003, 0.070]). Therefore, H4 was partially supported.

**Figure 2:**
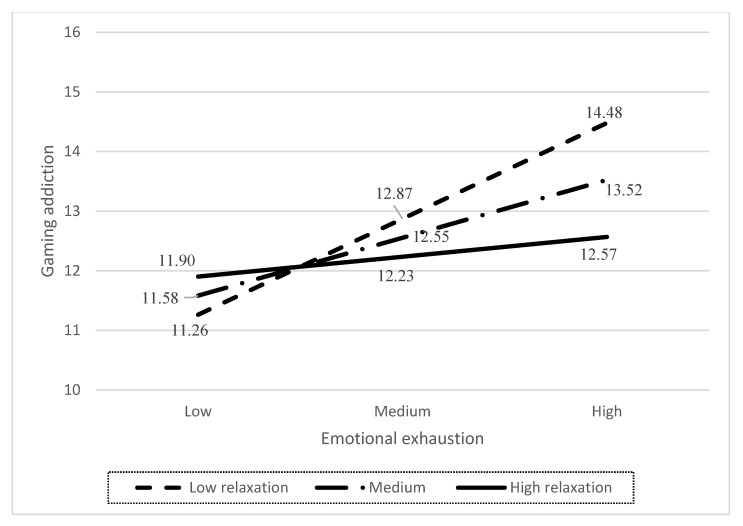
The illustration of the buffering effect of relaxation experience. *Note: Means of three groups in relaxation are 14.81, 17.22, and 19.63, respectively.*

The moderating effect of each type of recovery experience on the relationship between emotional exhaustion and gaming addiction is shown in Table 3. Among the four types of recovery experience, relaxation had a significant moderating effect on the relationship between emotional exhaustion and gaming addiction (β = −0.05, 95%CI [−0.09, −0.01]). As illustrated in Figure 2, the positive relationship was significant at low level of relaxation (β = 0.26, 95% CI [0.11, 0.42]) but insignificant at high level of relaxation (β = 0.03, 95% CI [−0.14, 0.19]). Therefore, H5 was partially supported.

## 4. Discussion

Gaming addiction has been recognized as a mental disorder that would interfere with individuals’ functioning and hamper their well-being. However, little was known about workers’ gaming addiction as well as its relation to emotional consequences and recovery experiences related to work stress. Consistent with the Conservation of Resources Theory [29] and Effort-Recovery Theory [31], this study is the first to provide empirical support regarding the protective effects of recovery experiences against gaming addiction as a result of workplace emotional exhaustion. 

As anticipated, we found emotional exhaustion to be a salient risk factor for Chinese workers’ poor mental health, including gaming addiction. This finding was consistent with the previous studies on addictive behaviors, including alcohol dependence, Internet addiction, and mobile phone addiction [45,46]. However, one should note that emotional exhaustion can also be an indicator of poor emotional regulation, which is both a cause and result of addictive behaviors [47,48]. Therefore, further studies are called for to explore not only whether emotional exhaustion longitudinally predicts gaming addiction but also whether the latter would increase one’s level of emotional exhaustion in working adults. In addition to emotional exhaustion, demographic risk factors of workers’ gaming addiction should also be noted. This study replicated the higher susceptibility of men to gaming addiction, compared to women, observed in other studies among both adolescent and adult samples [49,50]. Meanwhile, we found that younger workers were more likely to have gaming addiction than older ones. One possible reason is that young people may be more attracted by online and offline games in the new technological devices (e.g., smartphones, touchscreen tablets, and video game consoles). 

In line with Effort-Recovery Model, three of the four types of recovery experiences were found to be negatively associated with emotional exhaustion, probably because they would compensate for resource depletion by the job strain [15]. The recovery effectiveness of these four types of recovery experiences varies [51], and this study showed that not all types of recovery experiences matter. In particular, control and mastery experiences showed significant negative correlations with emotional exhaustion of Chinese working adults. It is plausible that those workers with more control and mastery experience may be more efficacious in choosing and utilizing leisure activities to reduce work-related stress and strains. 

To our surprise, psychological detachment had a weak but positive effect on emotional exhaustion. This finding is not consistent with meta-analytic evidence regarding the association between the lack of detachment and mental problems or poor work performance [52]. Lu et al. suggested that workers who value social or organizational goals and approval are less likely to benefit from detachment experience for improving work performance, probably because these values may induce cognitive dissonance or conflicts with their cognitions and behaviors related to detaching from work [53]. Therefore, we ponder that, under the collectivistic culture, detachment experience might not effectively protect and even mildly hamper our Chinese working participants’ mental health. The replicability of the current findings and the underlying mechanisms speculated must be further investigated in future cross-cultural research with cultural factors such as social-oriented values and motivation also tested.

In addition to its negative bivariate correlation with gaming addiction, control experience was also found to have a protective indirect effect against gaming addiction via lowering emotional exhaustion. Control experience occurs when people can arrange their leisure time and activities with autonomy. Such recovery experience facilitates the building up of internal resources (e.g., by increasing a sense of self-efficacy) and hence provides extra defense against negative emotional and mental strain [15]. Our findings not only provide additional evidence that control experience is negatively associated with psychiatric problems (e.g., sleep disturbance and depressive symptoms [54]) but also a potential psychological mechanism underlying gaming addiction development. Considering the negative correlation observed between control experiences and work hours in this study, organizations must set and adhere to the maximum work hour policy. They should encourage employees to participate in off-work activities voluntarily and at their own pace. Doing so can facilitate employees’ control over their leisure and thus recovery from resource loss during work hours. 

Relaxation experience, which resulted from the positive effects of the state of being free from tension and worry, also showed a significant negative association with gaming addiction among our Chinese workers. It may help keep both the body and mind at a low activation level, which allows their physiological and psychological recovery from the resource loss due to work [15]. Consistent with the previous finding of the negative association between relaxation experience and depression/anxiety symptoms [36], our findings suggest that people with such experience are less likely to suffer from gaming addiction. Consistent with our findings, a case study on the effectiveness of cognitive-behavioral therapy suggests that relaxation techniques can help patients cope with a craving for not only alcohol use but also gaming [55]. Future research may investigate the interrelations of relaxation experience, craving control, gaming addiction, and its comorbid disorders (i.e., depression/anxiety) for a better understanding of how recovery experience of relaxation lower workers’ susceptibility to gaming addiction [56,57]. 

Last but not least, as hypothesized, relaxation experience buffered the effect of emotional exhaustion on gaming addiction, with the strength of association between emotional exhaustion and gaming addiction decreasing at higher levels of relaxation experience. The finding is consistent with the Effort-Recovery Model [31] and another study on cancer workers regarding their relaxation experience and reduced risk of emotional exhaustion (e.g., depression, restlessness, and hopelessness) [33]. This study extended this line of research by showing the buffering effects of recovery experiences on the risk of emotional exhaustion on addictive disorders. During the recovery process, it is plausible that positive emotions are generated from relaxation experiences, which may help workers to handle their emotional exhaustion from work and hence weaken its negative effects on gaming addiction. Therefore, workshops for relaxation techniques and activities (e.g., yoga and painting) can be incorporated into workplace-based prevention programs for mental distress and disorders.

Our findings should be cautiously interpreted given some limitations of this study. First, the cross-sectional study design is not allowed to test the causal relationships between emotional exhaustion, recovery experiences, and gaming addiction. Longitudinal and experimental designs should be considered in future studies. Second, gaming addiction was conceptualized and assessed as a continuous propensity instead of a clinical diagnosis in this study. We used a self-reported scale for its measurement, which has no validated diagnostic cutoff, and no data on the clinical diagnosis of the participants was obtained. Finally, data were collected online, and this might limit the sample representativeness (e.g., 58% female participants) and thus the generalizability of the findings. 

## 5. Conclusions

This study made a suitable contribution to the literature because this study not only identified the risk effects of some demographics (i.e., younger age and males) and emotional exhaustion on working adults’ gaming addiction but also is the first to investigate the potential protective effects and mechanisms of recovery experiences against gaming addiction. Our findings showed that two out of the four recovery experiences (i.e., relaxation and control) had a significant and negative relationship with the gaming addiction of working adults. Moreover, further analyses revealed two mechanisms of the protective effects of recovery experiences: control experience had a negative indirect effect on gaming addiction by lowering emotional exhaustion due to work, while relaxation experience attenuated the risk effect of emotional exhaustion on gaming addiction. Interventions targeted to increase the workers’ awareness of their recovery experiences (particularly of relaxation and control) and to improve recovery opportunities are recommended to lower the risk of gaming addiction and even the negative effects of emotional exhaustion. 

## Figures and Tables

**Table 1:** Descriptive statistics and correlations among variables (N = 205).

	M	SD	1	2	3	4	5	6	7	8	9	10
1. Gaming addiction	12.63	4.46	--									
2. Recovery experience-Detachment	10.66	5.04	0.03	--								
3. Recovery experience-Relaxation	17.22	2.41	−0.16 *	0.29 ***	--							
4. Recovery experience-Mastery	15.61	3.14	−0.07	−0.12	−0.07	--						
5. Recovery experience-Control	17.32	2.77	−0.18 **	0.11	0.39 ***	0.07	--					
6. Emotional exhaustion	11.15	5.42	0.24 ***	0.15 *	−0.13 #	−0.14 *	−0.41 ***	--				
7. Work hours	45.00	10.39	0.03	−0.10	−0.13	0.08	−0.16 *	0.07	--			
8. Educational level	4.92	0.60	−0.06	0.05	0.04	0.11	−0.01	0.14	−0.14	--		
9. Age ^	3.94	1.46	−0.18 **	−0.23 ***	0.03	0.02	−0.05	−0.06	0.10	−0.15 *	--	
10. Sex ^^	--	--	−0.19 **	0.05	0.05	−0.16 *	0.02	−0.07	−0.08	0.10	−0.06	--

*Note*: # *p* = 0.07; * *p* < 0.05; ** *p* < 0.01; *** *p* < 0.001; ^: Ranging 1 (i.e., ≤20 years) to 8 (i.e., ≥51 years); ^^: 1 = Male, 2 = Female.

**Table 2:** Testing the indirect effects of recovery experiences on gaming addiction.

	Direct Effect Size [95%CI]	Indirect Effect Size [95%CI]
1. Detachment	0.003 [−0.124, 0.963]	0.031 [0.003, 0.077]
2. Relaxation	−0.169 [−0.441, 0.224]	−0.010 [−0.083, 0.041]
3. Mastery	−0.028 [−0.219, 0.764]	−0.024 [−0.078, 0.021]
4. Control	−0.113 [−0.362, 0.370]	−0.128 [−0.241, −0.034]

**Table 3:** Testing the moderating effects of recovery experiences on the relationship between emotional exhaustion and gaming addiction.

Interaction Effect	R^2^_change_	F_change_	*p*	Interaction Effect Size	95%CI
1. Emotional exhaustion × Detachment	<0.001	0.06	0.80	0.002	[−0.02, 0.02]
2. Emotional exhaustion × Relaxation	0.02	5.08	0.02	−0.05	[−0.09, −0.01]
3. Emotional exhaustion × Mastery	<0.001	0.06	0.81	−0.003	[−0.12, 0.13]
4. Emotional exhaustion × Control	0.003	0.58	0.45	−0.003	[−0.13, 0.12]

## Data Availability

The datasets used and/or analyzed during the current study are available from the corresponding author upon reasonable request.
